# Supplementary material for: Identification and analysis of immunoreactive proteins of Shigella flexneri in human sera and stool specimens
Source: PeerJ. 2024 May 28;12:e17498. doi: 10.7717/peerj.17498 (PMC11141557; doi:10.7717/peerj.17498)
Supplement: Supplemental Information 1 [file peerj-12-17498-s001.pdf]

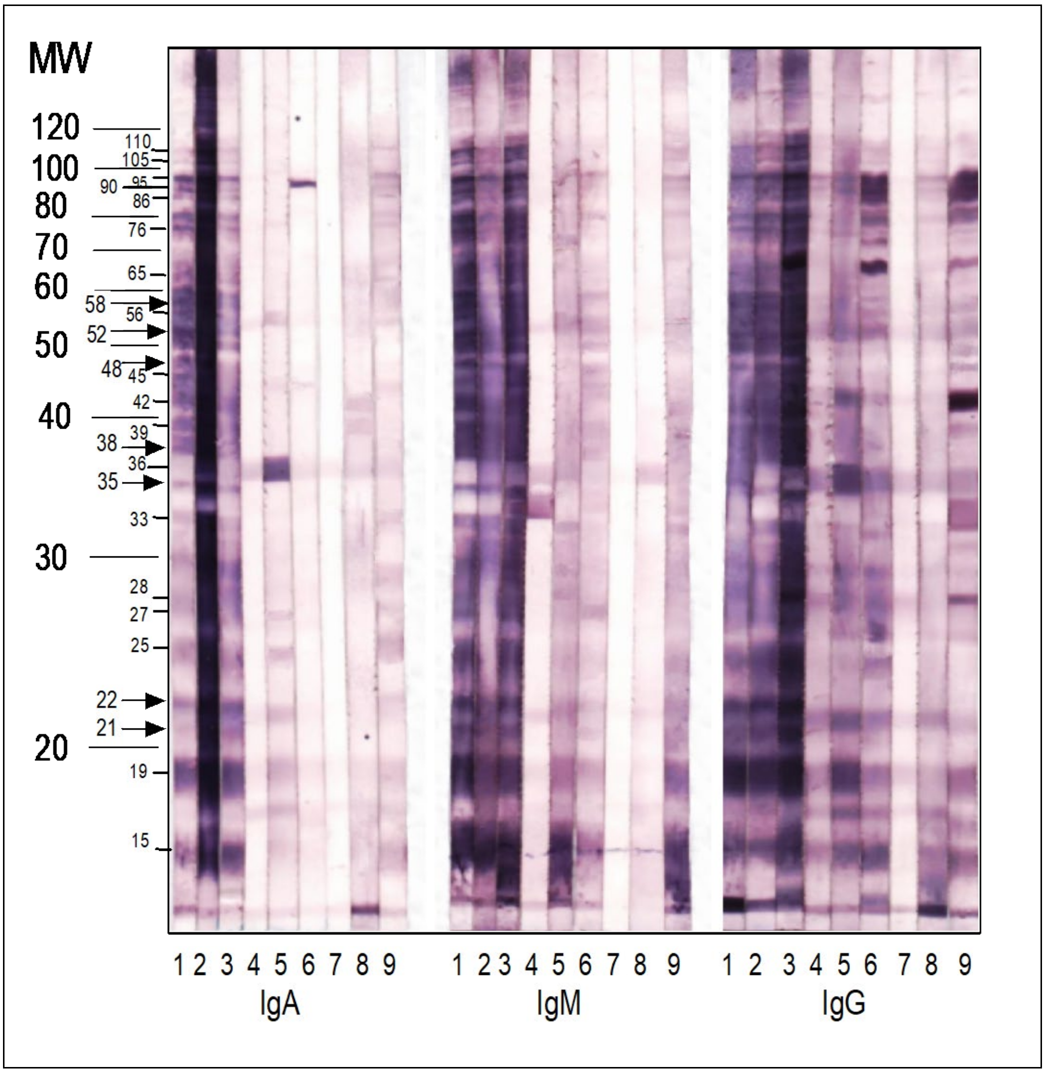

Supplementary figure S1. The response of *Shigella flexneri* infection sera compared with other infection sera against the surface protein of *Shigella flexneri* strain SF480. Lanes 1 to 9 show the response of *Shigella flexneri* infection serum and other infection serum to the surface protein of *Shigella flexneri* strain SF480. Lane 1: *Shigella flexneri* SF100 infection serum, lane 2: *Shigella flexneri* SF480 infection serum, lane 3: *Shigella flexneri* SF262 infection serum, lane 4: enteropathogenic *Escherichia coli* infection serum 1, lane 5: enteropathogenic *Escherichia coli* infection serum 2, lane 6: *Campylobacter jejuni* infection serum, lane 7: *Vibrio cholerae* infection serum, lane 8: *Salmonella typhi* infection serum, lane 9: *Salmonella paratyphi* A infection serum.

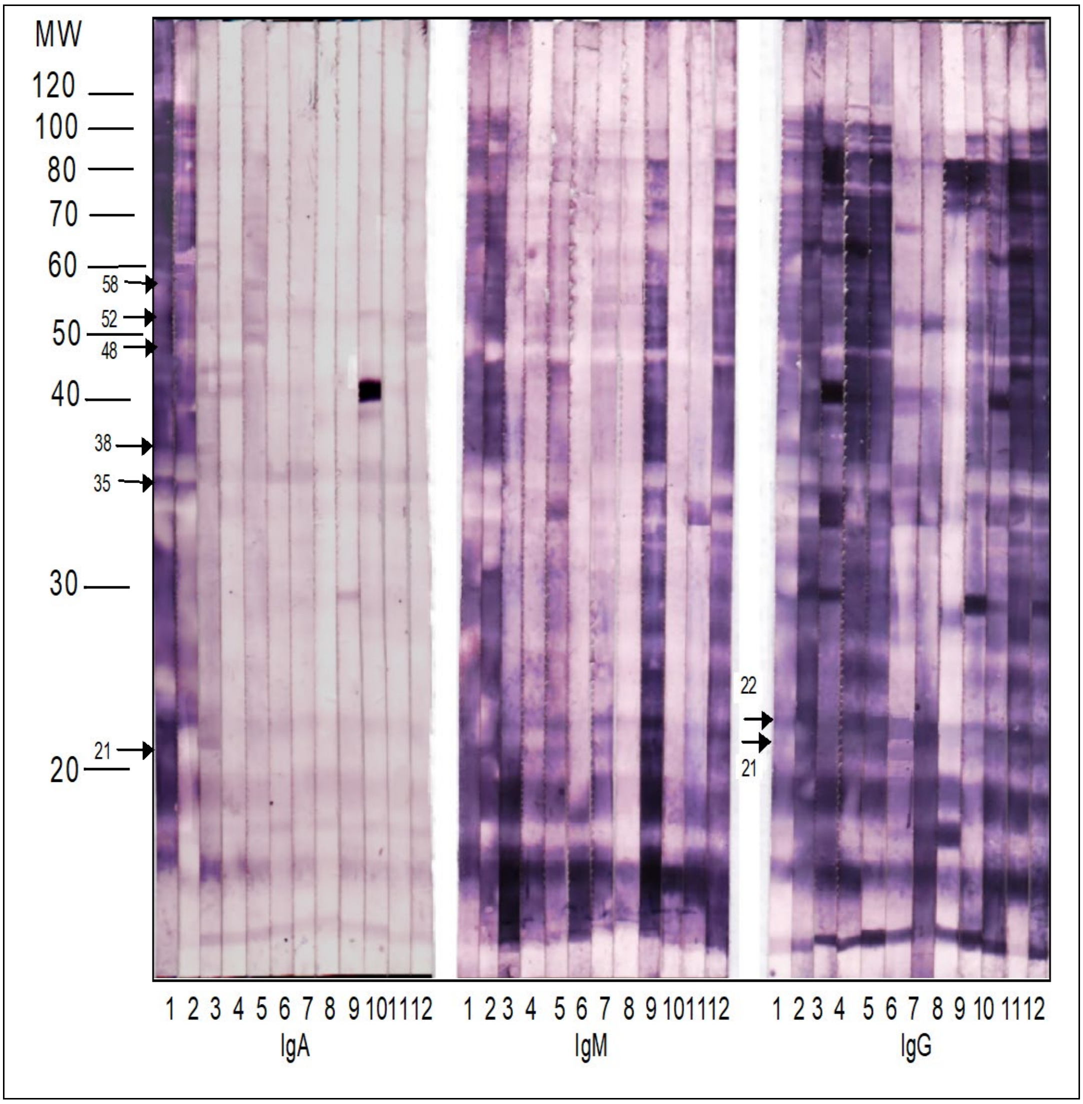

Supplementary figure S2. The response of *Shigella flexneri* infected serum compared with normal human serum to the surface protein of *Shigella flexneri* strain SF480. Lanes 1 to 11 show the reaction of *Shigella flexneri* infected serum and normal human serum against the surface protein of *Shigella flexneri* strain SF480. Lane 1: *Shigella flexneri* SF480 infection serum, lane 2: *Shigella flexneri* infection serum (pooled), lane 3: normal human serum N1, lane 4: normal human serum N2, lane 5: normal human serum N3, lane 6: normal human serum N4, lane 7: normal human serum N5, lane 8: normal human serum N6, lane 9: normal human serum N7, lane 10: normal human serum N8, lane 11: normal human serum N9 and lane 12: normal human serum N10.

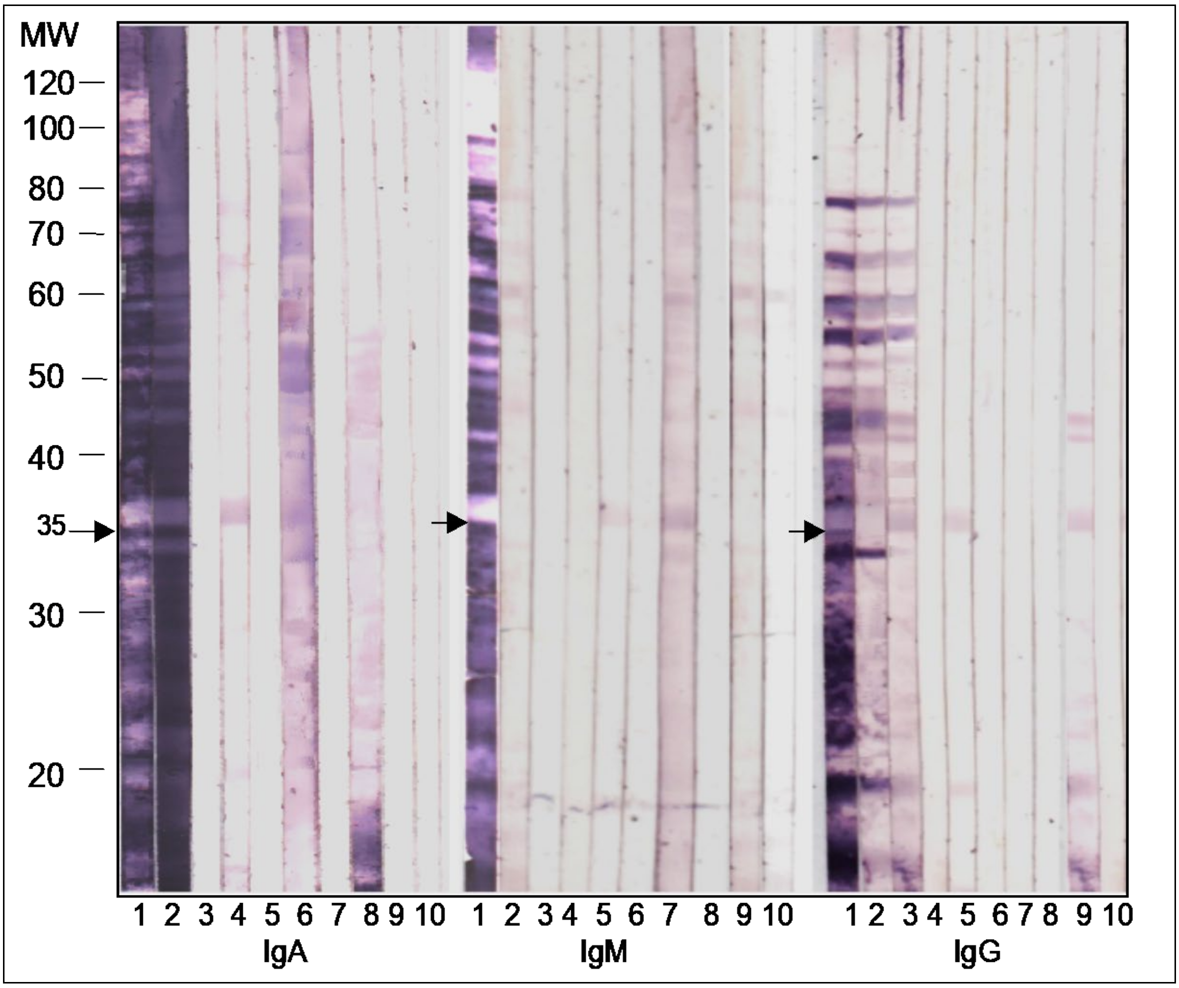

Supplementary figure S3. The response of stool suspensions of *Shigella flexneri* infection compared with stool suspensions of other infections against the surface protein of *Shigella flexneri* strain SF480. Lanes 1 to 7 show the response of serum and stool suspensions of *Shigella flexneri* infection compared to stool suspensions of other infections against the surface protein of *Shigella flexneri* strain SF480. Lane 1: *Shigella flexneri* SF480 infection serum, lane 2: *Shigella flexneri* SF10 infection stool suspension, lane 3: enteropathogenic *Escherichia coli* infection stool suspension 1, lane 4: *Salmonella typhi* infection stool suspension 1, lane 5: *Salmonella typhi* infection stool suspension 2, lane 6: stool suspension of *Vibrio cholerae* infection, lane 7: stool suspension of *Campylobacter jejuni* infection, lane 8: stool suspension of enteropathogenic *Escherichia coli* 2 infection, lane 9: stool suspension of Entamoeba histolytica infection, lane 10: stool suspension of *Giardia lamblia* infection.
